# Response to White and Lewis: Letter to editor in response to Has *Chlamydia trachomatis* prevalence in young women in England, Scotland and Wales changed? Evidence from national probability surveys. *Epidemiology and Infection. 2019*

**DOI:** 10.1017/S0950268819001560

**Published:** 2019-09-20

**Authors:** D. Z. Kounali, A. E. Ades, K. Soldan, P. Horner

**Affiliations:** 1 Population Health Sciences, Bristol Medical School, 38 Whatley Road Bristol BS2 8PS, UK; 2 National Institute for Health Research Health Protection Research Unit in Evaluation of Interventions, University of Bristol, Bristol, UK; 3 Public Health England, 61 Colindale Avenue, London NW9 5EQ, UK

White and Lewis [1] comment on our article [2] highlighting the methodological issues arising when attempting to use the National survey of Attitudes and Sexual Lifestyles (NATSAL) to calibrate estimates of seroprevalence derived from data available by sources such as the PHE Seroepidemiology Unit [3] and Health Survey for England [4]. White and Lewis [1] do not challenge our observations. We agree with White and Lewis [1] on the importance of data on health-seeking behaviour. It is not possible to use data on individuals who are tested for CT to make inferences about CT prevalence, or changes in CT prevalence over time, without information on how the CT prevalence relates to the probability of being tested, and how that changes over time [5–7]. Individuals may be tested for a number of reasons: following an *ad hoc* offer of opportunistic testing; as a result of symptoms; or concern about recent sexual encounters. Each of these factors may impact on CT prevalence among those tested in GP surgeries or GUM clinics.
